# 14-3-3ε augments OGT stability by binding with S20-phosphorylated OGT

**DOI:** 10.1016/j.jbc.2024.107774

**Published:** 2024-09-12

**Authors:** Sheng Yan, Kemeng Yuan, Xinyi Yao, Qiang Chen, Jing Li, Jianwei Sun

**Affiliations:** 1 Beijing Key Laboratory of DNA Damage Response and College of Life Sciences, Capital Normal University, Beijing, China; 2 Yunnan Key Laboratory of Cell Metabolism and Diseases, State Key Laboratory for Conservation and Utilization of Bio-Resources in Yunnan, Center for Life Sciences, School of Life Sciences, Yunnan University, Kunming, China; 3 Department of Gastrointestinal Surgery, Medical Research Institute, Zhongnan Hospital of Wuhan University, Frontier Science Center for Immunology and Metabolism, Wuhan University, Wuhan, China

**Keywords:** O-GlcNAcylation, OGT, cytokinesis, uterine carcinoma, Chk1, 14-3-3

## Abstract

The relationship between O-linked N-acetylglucosamine (O-GlcNAc) transferase (OGT) and mitosis is intertwined. Besides the numerous mitotic OGT substrates that have been identified, OGT itself is also a target of the mitotic machinery. Previously, our investigations have shown that Checkpoint kinase 1 (Chk1) phosphorylates OGT at Ser-20 to increase OGT levels during cytokinesis, suggesting that OGT levels oscillate as mitosis progresses. Herein we studied its underlying mechanism. We set out from an R17C mutation of OGT, which is a uterine carcinoma mutation in The Cancer Genome Atlas. We found that R17C abolishes the S20 phosphorylation of OGT, as it lies in the Chk1 phosphorylating consensus motif. Consistent with our previous report that pSer-20 is essential for OGT level increases during cytokinesis, we further demonstrate that the R17C mutation renders OGT less stable, decreases vimentin phosphorylation levels and results in cytokinesis defects. Based on bioinformatic predictions, pSer-20 renders OGT more likely to interact with 14-3-3 proteins, the phospho-binding signal adaptor/scaffold protein family. By screening the seven isoforms of 14-3-3 family, we show that 14-3-3ε specifically associates with Ser-20-phosphorylated OGT. Moreover, we studied the R17C and S20A mutations in xenograft models and demonstrated that they both inhibit uterine carcinoma compared to wild-type OGT, probably due to less cellular reproduction. Our work is a sequel of our previous report on pS20 of OGT and is in line with the notion that OGT is intricately regulated by the mitotic network.

O-linked N-acetylglucosamine (O-GlcNAc) transferase (OGT) is the only enzyme that catalyzes all monosaccharide O-GlcNAcylation reactions in the cell (1, 2). During the cell division, OGT is dynamically regulated at both the protein level and the post-translational level (3, 4). Specifically, Checkpoint-kinase 1 (Chk1) phosphorylates OGT at Ser-20 to upregulate OGT abundance during cytokinesis, the abrogation of which will lead to the vimentin bridge phenotype during cytokinesis (5). This mechanism is conserved in gut homeostasis in *Drosophila* and the DNA damage response in mouse embryonic stem cells and mouse embryonic fibroblasts (6), suggesting that studying critical residues of OGT will reveal fundamental mechanisms of glycosylation in distinct biological processes (7). In another study, Ser-20 is found to be phosphorylated by calcium/calmodulin-dependent kinase II to promote O-GlcNAcylation in liver autophagy (8), again highlighting the importance of pSer-20.

Although much has been learned about the 7000 OGT substrates in the last 4 decades, comparably less has been reported on post-translational modifications (PTMs) on OGT itself. Glycogen synthase kinase 3b (GSK3β) phosphorylates OGT at Ser-3/Ser-4 and upregulates OGT activity (9); OGT itself is O-GlcNAcylated at Ser-389, in close proximity to its nuclear localization signal (^451^DFP^453^) (10); OGT is phosphorylated by Adenosine-monophosphate-activated protein kinase (AMPK) at Thr-444 and inhibits OGT-chromatin association (11, 12); Epidermal growth factor (EGF) stimulates OGT phosphorylation at Tyr-976 to promote the interaction between OGT and pTyr-binding proteins (13). These and potentially other PTMs may underline how OGT responds to environmental stress and confer specificity to OGT interaction partners (7). After all, “every amino acid matters” (14).

Previously, we showed that Chk1-mediated OGT phosphorylation at Ser-20 localizes to the midbody, which stabilizes OGT, promotes vimentin pSer-71 and subsequent vimentin filament severing during the cytokinesis stage (5). In this report, we continued our above work on OGT pSer-20. The Cancer Genome Atlas (TCGA) reveals that the OGT-R17C mutation is implicated in uterine carcinoma. As Arg-17 lies right at the consensus motif of Chk1 phosphorylation, we investigated its potential effect on pSer-20. We found that R17C abolished Ser-20 phosphorylation and affected vimentin phosphorylation. By xenograft assays, we found that both R17C and S20A affected the mitotic process and inhibited tumorigenesis. Both bioinformatic predictions and our mutagenesis screening suggest that pS20-OGT associates with 14-3-3ε, one of the seven members of the phospho-binding 14-3-3 adapter protein family (15). Our data extend our previous investigation on pSer-20 and suggest that PTMs on OGT are functionally relevant to human diseases.

## Results

### The R17C mutation of OGT abolishes S20 phosphorylation

**Figure 1:**
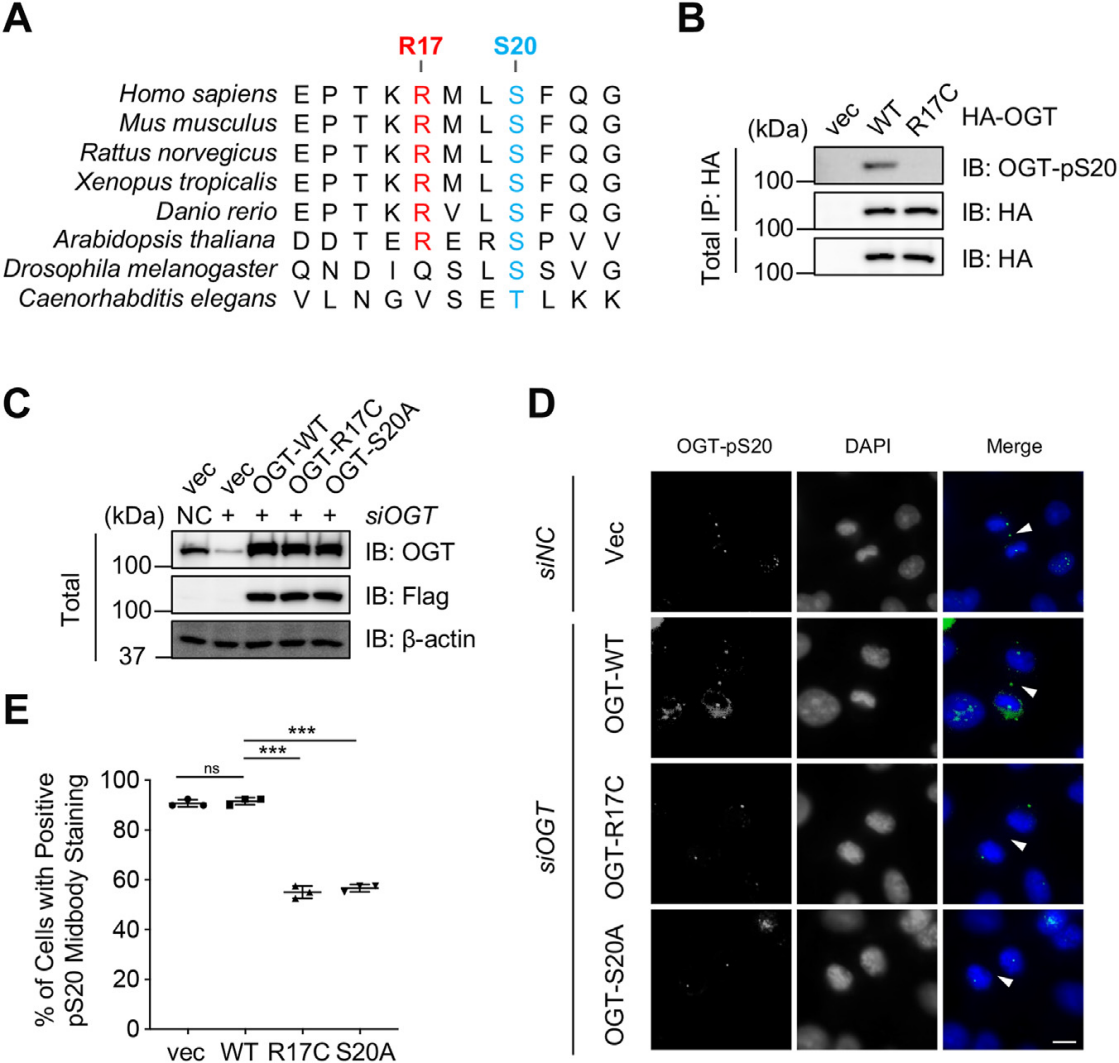
**The R17C mutation of OGT abolishes pS20**. *A*, Arg17 and Ser20 of OGT are conserved. *B*, HEK-293T cells were transfected with HA-OGT-WT or -R17C, and then the lysates were immunoprecipitated and immunoblotted with the HA antibody and rabbit anti-OGT-pS20 antibody as indicated. *C*, Western blotting analyses of OGT expression levels in OGT-WT, OGT-S20A, or OGT-R17C stable transfected cells treated with control siRNA or *siOGT*. *D*, cells in (*C*) were synchronized to the cytokinetic stage, and were then stained with OGT-pS20 antibodies. Arrowheads indicate staining at the midbody. Scale bar represents 10 μm. *E*, quantitation of cells with positive OGT-pS20 signals from three independent experiments. More than 40 anaphase cells were counted in each experiment. The statistical analysis in (*E*) was performed using Student’s *t* test. ns, nonspecific; ∗∗∗*p* < 0.001.

We noted that the R17C mutation of OGT is involved in uterine carcinoma in TCGA, therefore we are interested in studying its underlying mechanism. When sequences were compared, we found that Arg-17 is relatively conserved among different species (Fig. 1*A*). As Arg-17 lies right in the consensus motif for Chk1 substrates, we suspected that it might affect pSer-20. We used the pSer-20 antibody previously manufactured from our lab (5), and found that indeed R17C completely abolished pSer-20 signals (Fig. 1*B*). To examine its effect cytologically, we generated cells stably expressing OGT-WT, -S20A and -R17C (Fig. 1*C*), and examined pSer-20 localization using immunofluorescence (Fig. 1*D*). pSer-20 antibodies consistently labeled the midbody in WT cells, but not in the S20A or R17C cells (Fig. 1, *D* and *E*). These results suggest that Arg-17 is essential for Chk1-mediated phosphorylation of OGT.

### OGT-R17C increases binding with Chk1

**Figure 2:**
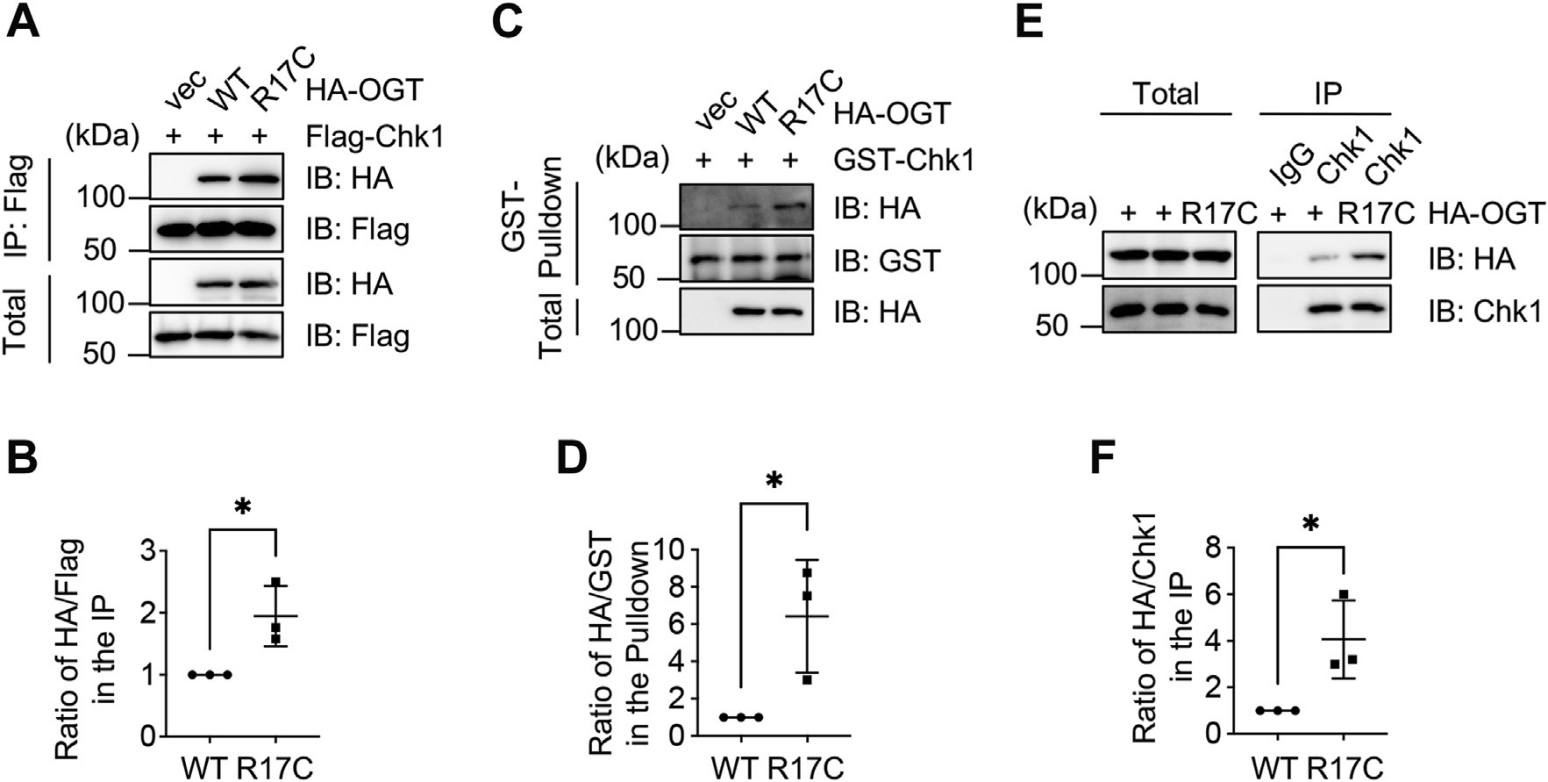
**The R17C mutation of OGT increases binding with Chk1**. *A*, HEK-293T cells were transfected with HA-OGT-WT, -R17C and Flag-Chk1, and then the lysates were immunoprecipitated and immunoblotted with the antibodies indicated. *B*, the quantitation of (*A*). *C*, cells were transfected with HA-OGT-WT and -R17C plasmids. Recombinant GST-Chk1 proteins were incubated with the cellular lysates, and GST pulldown experiments were carried out. *D*, the quantitation of (*C*). *E*, OGT-R17C increases binding with endogenous OGT. *F*, the quantitation of (*E*). The statistical analysis in (*B*), (*D*) and (*F*) was performed using Student’s *t* test. ∗*p* < 0.05.

As phosphorylation generally involves the transient interaction between the kinase and the substrate, we measured the binding between OGT-R17C and Chk1. Exogenously expressed OGT-R17C increased affinity with Flag-Chk1 (Fig. 2, *A* and *B*), suggesting that Chk1 associates with OGT but cannot phosphorylate OGT, resulting in elevated interaction. This is consistent with previous observations that the kinase-dead mutant of the kinase shows higher affinity with the substrates (16).

We also examined GST-pulldown and endogenous interactions between OGT and Chk1 (Fig. 2, *C*–*F*), and the data consistently showed increased binding between OGT-R17C and recombinant (Fig. 2*C*) or endogenous (Fig. 2*E*) Chk1. These results suggest that the Arg-17 mutation does not affect the initial interaction between OGT and Chk1. Chk1 binds the OGT mutant, but cannot phosphorylate it, therefore OGT-R17C lingers longer than OGT-WT.

### The R17C mutation reduces OGT stability

**Figure 3:**
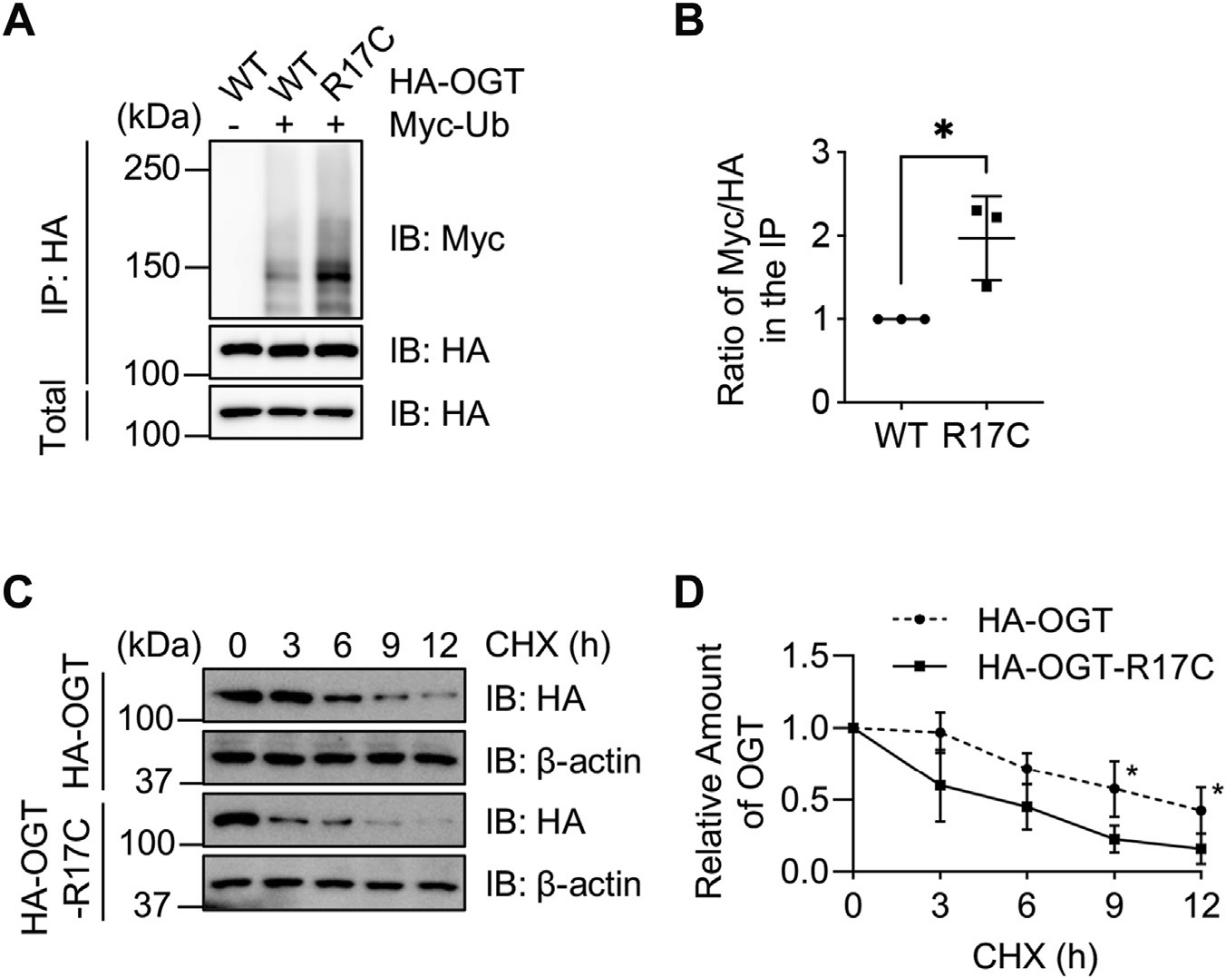
**The R17C mutation destabilizes OGT**. *A*, HEK-293T cells were transfected with HA-OGT-WT, -R17C and Myc-Ub, and then the lysates were immunoprecipitated and immunoblotted with the antibodies indicated. *B*, the quantitation of (*A*) using Student’s *t* test. *C* and *D*, cycloheximide (CHX) pulse-chase assays. Cells were transfected with HA-OGT-WT or HA-OGT-R17C plasmids, then treated with CHX for different durations. A two-way ANOVA test was used for statistical analysis in (*D*). ∗ indicates *p* < 0.05.

We have previously shown that pSer-20 stabilizes OGT by decreasing proteasome-mediated degradation (5), therefore we wondered whether R17C also has the same effect. To this end, HA-OGT was co-transfected with Myc-Ub, and OGT ubiquitination levels were evaluated (Fig. 3, *A* and *B*). As expected, R17C upregulates ubiquitination compared to the WT. When cycloheximide (CHX) pulse-chase experiments were carried out, OGT-WT has a longer half-life than R17C (Fig. 3, *C* and *D*). These results are in line with our previous findings that pSer-20 increases OGT abundance (5) and pS20 correlates with cellular O-GlcNAcylation levels (8).

### The R17C mutation phenocopies the S20A mutant in vimentin bridge formation

**Figure 4:**
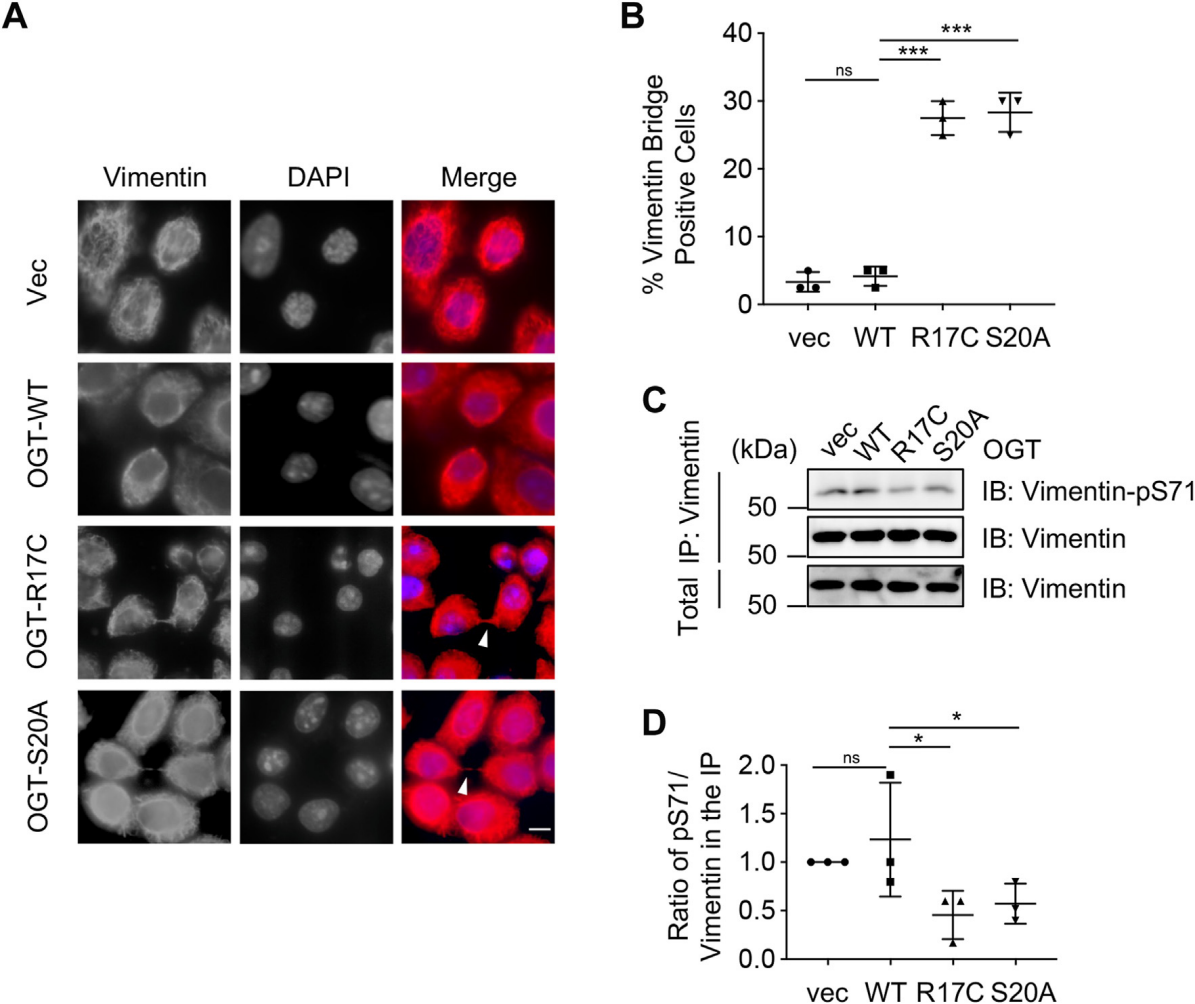
**The R17C mutant results in vimentin bridges during cytokinesis**. *A*, OGT stable transfected HeLa cells were synchronized to the cytokinetic stage, and were then stained with the vimentin antibody, together with DAPI. Arrowheads indicate staining at the vimentin bridges. Scale bar, 10 μm. *B*, HeLa cells were quantitated for vimentin bridges, which indicate defects in cytokinesis. The experiemnts were repeated for 3 times, with more than 40 anaphase cells counted in each experiment. *C*, OGT stable transfected HeLa cells were synchronized to the cytokinetic stage. The lysates were immunoprecipitated and immunoblotted with the antibodies as indicated. *D*, the quantitation of (*C*). The statistical analysis in (*B*) and (*D*) was performed using Student’s *t* test. ns, nonspecific; ∗*p* < 0.05; ∗∗∗*p* < 0.001.

As we demonstrated that OGT-S20A mutants decreased vimentin pSer-71 levels and impaired vimentin bridge severing during cytokinesis (5), we wondered whether the same holds true for R17C. Stable transfectants were synchronized to cytokinesis and stained for vimentin antibodies (Fig. 4, *A* and *B*). WT cells showed separated cytokinetic cells, but vimentin bridges were discernable in both R17C and S20A cells (Fig. 4, *A* and *B*). We also used synchronized cells to test the vimentin-pSer-71 levels (Fig. 4, *C* and *D*), which were attenuated in the R17C and S20A cells.

### OGT-pS20 promotes stability by binding with 14-3-3ε

We wondered what is the underlying mechanism that stabilizes S20-phosphorylated OGT. According to http://www.compbio.dundee.ac.uk/1433pred (17), pS20 is predicted to be one of the highest hits for 14-3-3 binding. The 14-3-3 protein family is highly conserved. They bind phospho-Ser/Thr proteins and take part in many signaling events in diverse biological processes (18, 19). Moreover, O-GlcNAcylated proteins have been shown to bind 14-3-3 directly (20), suggesting that 14-3-3 may be associated with both OGT and its substrates.

**Figure 5:**
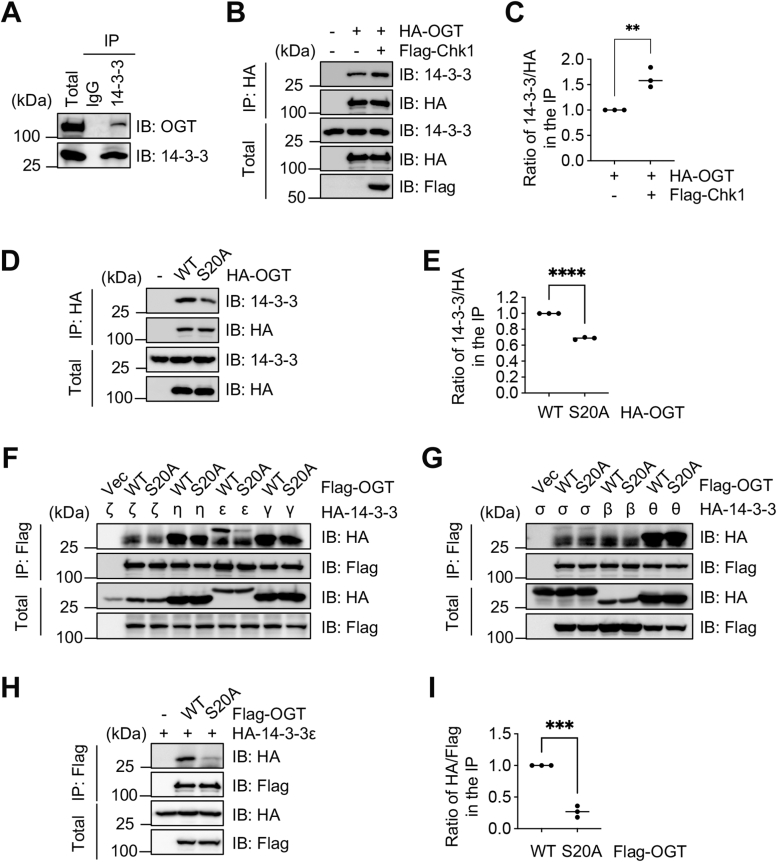
**14-3-3ε specifically binds S20-phosphorylated OGT**. *A*, co-IP between endogenous OGT and 14-3-3. *B*, cells were transfected with HA-OGT and Flag-Chk1. The lysates were immunoprecipitated with anti-HA antibodies and immunoblotted with anti-HA and anti-pan 14-3-3 antibodies as indicated. *C*, the quantitation of (*B*). *D*, cells were transfected with HA-OGT-WT and HA-OGT-S20A, and analyzed as in (B). *E*, the quantitation of (*D*). *F–G*, cells were transfected with Flag-OGT-WT, Flag-OGT-S20A, HA-14-3-3-ζ, -η, -ε, and -γ plasmids in (*F*), and HA-14-3-3-σ, -β, and -θ plasmids in (*G*). *H*, cells were transfected with Flag-OGT-WT, Flag-OGT-S20A and HA-14-3-3ε plasmids. The lysates were immunoprecipitated with anti-Flag antibodies and immunoblotted with the antibodies indicated. *I*, the quantitation of (*H*). The statistical analysis in (*C*), (*E*) and (*I*) was performed using Student’s *t* test. ∗∗*p* < 0.01; ∗∗∗*p* < 0.001; ∗∗∗∗*p* < 0.0001.

We first used a pan-14-3-3 antibody and found that endogenous OGT binds 14-3-3 (Fig. 5*A*). Then we tested the effect of Chk1 overproduction (Fig. 5, *B* and *C*), and found that Chk1 overproduction increases the association between exogenous OGT and 14-3-3. When we used the OGT-S20A mutant in the same assay (Fig. 5, *D* and *E*), S20A significantly attenuated the affinity between OGT and 14-3-3.

We then attempted to identify the exact isoform of 14-3-3 that interacts with S20-phosphorylated OGT. To this end, the seven isoforms of 14-3-3 (β/α, γ, ε, ζ, η, θ, σ) were co-transfected with OGT-WT and -S20A plasmids, and their interaction was assessed. As shown in Fig.5, F and G, OGT is associated with all seven isoforms. But only 14-3-3ε specifically binds with OGT-WT, not -S20A. We then confirmed the results by quantitation (Fig. 5, *H* and *I*). In sum, 14-3-3ε specifically binds with S20-phosphorylated OGT.

### Endogenous 14-3-3ε interacts with S20-phosphorylated OGT

**Figure 6:**
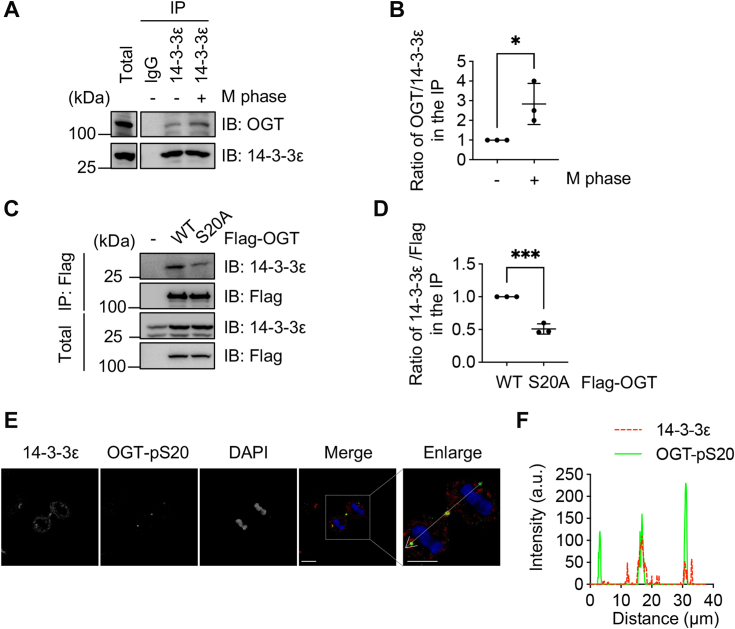
**Endogenous 14-3-3ε interacts with S20-phosphorylated OGT**. *A*, the cells were treated with Nocodazole to synchronize the cells to the M phase, and then the co-IP between endogenous OGT and 14-3-3ε was performed. *B*, quantitation of (*A*). *C*, cells were transfected with Flag-OGT-WT or Flag-OGT-S20A plasmids, then coIP experiments were performed. *D*, quantitation of (*C*). *E*, cells were synchronized to the cytokinetic stage, then stained with OGT-pS20 and 14-3-3ε antibodies and then examined under a Zeiss LSM780 confocal laser scanning microscopy. More than 30 synchronized cells were photographed. *F*, colocalization between OGT and 14-3-3ε was analyzed by ImageJ and GraphPad Prism. The scale bars in both the merge and enlarged images represent 10 μm. The statistical analysis in (*B*) and (*D*) was performed using Student’s *t* test. ∗*p* < 0.05; ∗∗∗*p* < 0.001.

To further study the interaction between 14-3-3ε and OGT, we used 14-3-3ε antibodies. As shown in Fig. 6, *A* and *B*, endogenous 14-3-3ε interacted with OGT in co-IP assays, and synchronization to the M phase increased the interaction. When the antibody was utilized to examine the association between 14-3-3ε and overproduced OGT, OGT-S20A showed marked down-regulation of the affinity with 14-3-3ε compared to OGT-WT (Fig. 6, *C* and *D*). These results suggest that 14-3-3ε interacts with S20-phosphorylated OGT.

As OGT-pS20 has been shown to localize to the midbody during cytokinesis (5), we are interested in finding out if 14-3-3ε has the same localization pattern. To this end, we used confocal microscopy to capture cells in cytokinesis and found that 14-3-3ε colocalizes with OGT-pS20 (Fig. 6, *E* and *F*). In sum, endogenous 14-3-3ε interacts with S20-phosphorylated OGT.

### The R17C and S20A mutations decrease tumorigenesis

**Figure 7:**
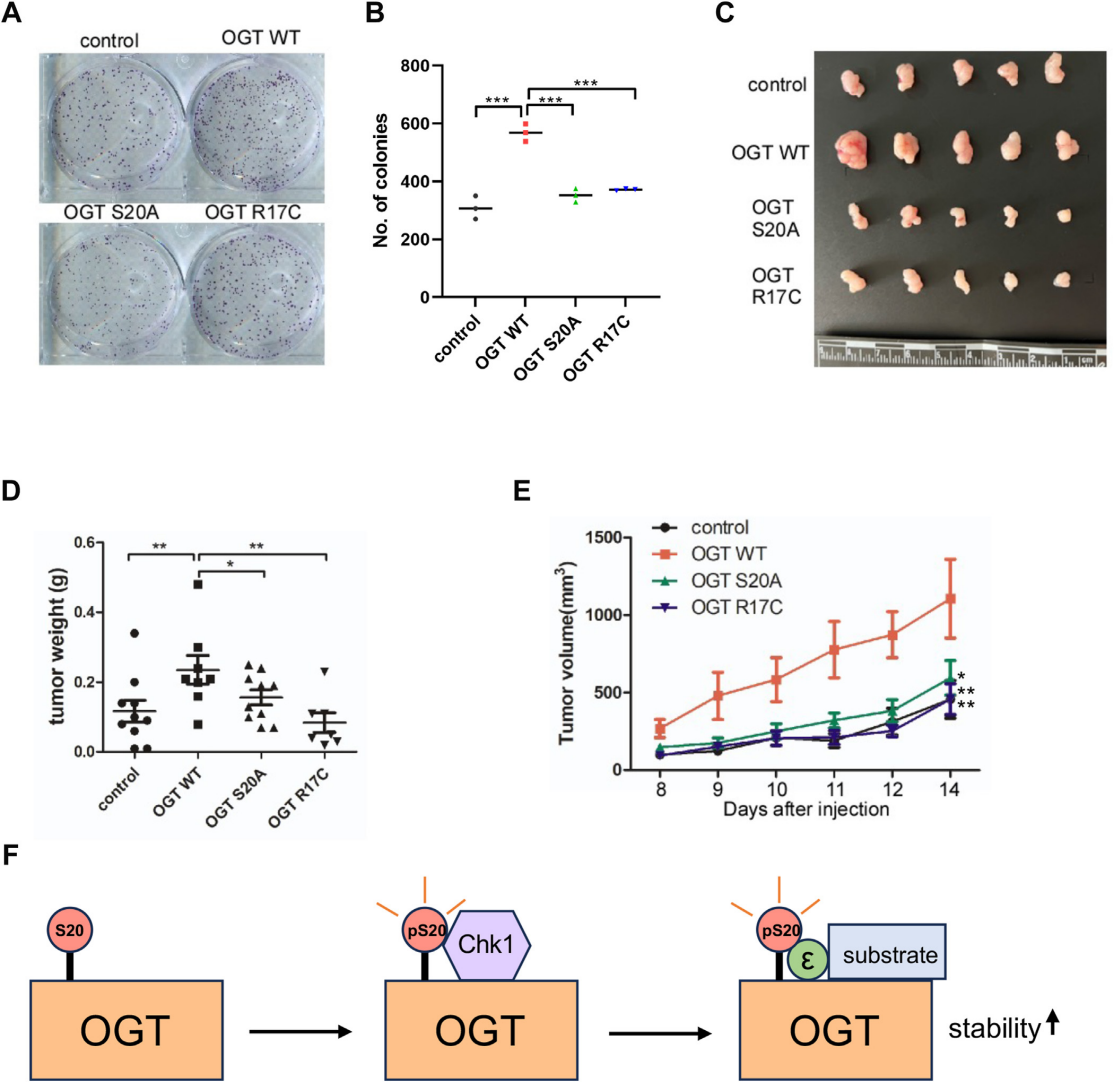
**R17C and S20A of OGT reduce cell proliferation and decrease tumorigenesis**. *A–B*, Colony formation assay showing that OGT-S20A and OGT-R17C mutants decrease cell proliferation. *C–E*, stable OGT-WT, OGT-S20A, OGT-R17C HeLa cells were injected into nude mice, and the tumors were photographed (C). Tumor sizes were quantitated in (*D*), and tumor weights were quantitated in (*E*). Quantitation was done with a one-way Anova. ∗*p* < 0.05; ∗∗*p* < 0.005; ∗∗∗*p* < 0.0005. *F*, a model depicting that Chk1 phosphorylates OGT at S20, creating a binding motif for 14-3-3ε, which bridges OGT and its substrates.

As all the above cytology and biochemical results suggest that the mutation of R17C exerts its function through pSer-20, we attempted to assess their functions *in vivo*. Colony formation assays were carried out, and R17C and S20A both decreased colony formation (Fig. 7, *A* and *B*), consistent with their cytokinetic defects (Fig. 4, *A* and *B*). We then used mouse xenograft experiments, and both the R17C and S20A mutants decreased tumor size and volume (Fig. 7, *C*–*E*). This is somewhat contradictory to the TCGA, and we think it might be due to impaired cell proliferation.

## Discussion

In this work, we demonstrate that OGT is a client protein of 14-3-3. In combination with our previous work, we show that Chk1-induced phosphorylation of OGT at S20 creates a binding motif for the phospho-protein binding 14-3-3. Specifically, 14-3-3ε binds with OGT-pS20 and stabilizes OGT (Fig. 7*F*). As previously 14-3-3 has been demonstrated to bind O-GlcNAcylated proteins directly (20), our results suggest that 14-3-3 may bridge OGT with its substrates, with pS20 being the critical link (Fig. 7*F*).

We found that OGT-R17C shows more robust interaction with Chk1 (Fig. 2). This may seem anti-intuitive at first sight. Due to the transient nature of the kinase-substrate interaction, the kinase usually associates with the substrate, phosphorylates it, and then departs. In the case of R17C, it is plausible that OGT-R17C interacts with Chk1, but they cannot disengage as Chk1 cannot phosphorylate OGT. Our observation is not unique, as the kinase-dead mutant of the kinase polo-like kinase 1 (PLK1) shows higher affinity with the substrates (16), because they cannot dissociate.

It has been a common theme that 14-3-3 binding will enhance protein stability. For instance, AMPK-induced phosphorylation of murine p27 at T197 (human T198) promotes binding between p27 and 14-3-3 and stabilizes p27 (21). Akt phosphorylates the tuberous sclerosis complex (TSC) subunit TBC1D7 and stabilizes it by 14-3-3 binding (22). We found all 7 14-3-3 isoforms interact with OGT (Fig. 5, *F* and *G*), but only ε shows specificity towards OGT-pS20. It is possible that other isoforms bind with OGT that is phosphorylated at other residues.

Acting on more than 7000 protein substrates, OGT has been known for its promiscuity and pleiotropic effects. Perhaps OGT partially confers its substrate specificity *via* 14-3-3. As 14-3-3 is known to form homodimers and heterodimers, it is possible that 14-3-3ε may partner with other 14-3-3 isoforms to connect OGT with many substrates, thus expanding the OGT interactome. Previous structural studies demonstrate that 14-3-3 may bind the O-GlcNAcylated Pro-Val-Ser/Thr motif through its amphipathic groove (20). We wonder whether the Arg17/Ser20 motif of OGT fits into that groove.

Being the only writer and reader for O-GlcNAc, both OGT and OGA levels have been under stringent control. OGT has been shown to be ubiquitinated by various ubiquitin E3 ligases, including X-linked inhibitor of apoptosis (XIAP) (23), LSD2 (24), Cullin5 (CUL5) (25), E6AP (26) and APC/C^Cdc20^ (27). It is deubiquitinated by deubiquitylating enzymes (DUBs) such as USP8 (28), BAP1 (29) and eukaryotic translation initiation factor three subunit H (EIF3H) (30). OGA is ubiquitinated by E3s, such as ubiquitin-protein ligase E3 module N-recognition five (UBR5) (31) and RBM14 (32). The E3s and DUBs acting on OGT could be many. It would be interesting in the future to find the exact E3 or DUB acting on or adjacent to pS20.

Functioning as the hub for many protein–protein interactions, 14-3-3 has been proposed as a promising therapeutic target for a wide range of diseases (19). The 14-3-3 molecular glue approach has many applications forthcoming. Considering the breadth and depth of OGT substrates, it would be worthwhile to develop glues targeting the 14-3-3ε/OGT complex.

## Experimental procedures

### Cell culture, antibodies

HeLa and HEK293T cells were purchased from ATCC. The cell lines were validated using STR profiling and free from *mycoplasma* contamination for all experiments. Antibodies were as follows: anti-Flag (Sigma, F1084), anti-GST (Gene Script, A00865), anti-HA (Bethyl, A190–108A), anti-IgG (Sigma, R2665), anti-Myc (PTM BIO, PTM-5390), anti-α-tubulin (MBL, PM054), anti-Chk1 (Santa Cruz Biotechnology, sc-56288), anti-β-actin (Sigma, A5441), anti-Vimentin (Santa Cruz Biotechnology, sc-373717), anti-Vimentin-pS71 (Abcam, ab115189), anti-OGT-pS20 (5), anti-OGT (Abcam, AB96718), 14-3-3ε (ThermoFisher Scientific, MA5-49207) and anti-pan 14-3-3 (Santa Cruz Biotechnology, sc-133233). si*OGT* sequences were described previously (5).

### IP and immunoblotting

Immunoprecipitation and immunoblotting experiments were performed as described before (5). The following primary antibodies were used for IB: anti-Flag (1:4000), anti-GST (1:2000), anti-HA (1:4000), anti-Myc antibody (1:2000), anti-α-tubulin antibody (1:1000), anti-Chk1 (1:1000), anti-β-actin (1:4000), anti-Vimentin (1:2000), anti-Vimentin-pS71 (1:1000), anti-OGT-pS20 (1:1000), anti-pan 14-3-3 (1:500), 14-3-3ε (1:1000). LAS-4000 was employed to detect signals and quantitated by the Multi Gauge software (Fujifilm).

### Confocal Immunofluorescence

For confocal immunofluorescence, dilutions of primary antibodies were 1:1000 for anti-14-3-3ε, and 1:1000 for anti-OGT-pS20 antibodies. Cell nuclei were stained with DAPI. After the slides were prepared, slides were photographed with a Zeiss LSM780 confocal laser scanning microscopy. We scanned the slides with 405 nm, 488 nm, and 594 nm lasers and 63 × oil objective lenses. After the images were obtained, ImageJ was used to process each channel of the image, and then the gray value of each channel is quantized along the direction of the arrow. Quantitation was drawn in the GraphPad Prism.

### Cell synchronization

Protocols to synchronize cells in the cytokinetic phase were described before (5). Briefly, cell cultures were first blocked by double thymidine, and collected 9 h after releasing from the second thymidine block.

### Colony formation assay

OGT-WT, OGT-S20A; OGT-R17C stable HeLa cells were collected by centrifugation after trypsin digestion of the logarithmic growth monolayer cells. Cell pellets were resuspended, counted, and diluted to 1 × 10ˆ3/ml. Cells were inoculated onto six-well plates at a density of 500 cells per well. 2 weeks later, cells were washed with PBS, fixed with 4% polyformaldehyde, and stained by 1 ml 0.1% crystal violet. And the colony numbers were counted.

### Mouse xenograft

OGT-WT, OGT-S20A; OGT-R17C stable HeLa cells were resuspended in Matrigel (Corning) and then injected into the flanks of nude mice (4–6 weeks old). Tumor volumes were measured from day 2 to 14 after injection. At 14 days after the injection, tumors were dissected. The mice were obtained from the Animal Research and Resource Center, Yunnan University (Certification NO. SCXK(Dian)K2021–0001). All animal work procedures were approved by the Animal Care Committee of the Yunnan University.

## Data availability

All data are contained in this manuscript.

## Conflict of interest

The authors declare that they have no conflicts of interest with the contents of this article.

## References

[1] Yang X., Qian K. (2017). Protein O-GlcNAcylation: emerging mechanisms and functions. Nat. Rev. Mol. Cell Biol..

[2] Hart G.W., Slawson C., Ramirez-Correa G., Lagerlof O. (2011). Cross talk between O-GlcNAcylation and phosphorylation: roles in signaling, transcription, and chronic disease. Annu. Rev. Biochem..

[3] Saunders H., Dias W.B., Slawson C. (2023). Growing and dividing: how O-GlcNAcylation leads the way. J. Biol. Chem..

[4] Liu C., Li J. (2018). O-GlcNAc: a sweetheart of the cell cycle and DNA damage response. Front. Endocrinol. (Lausanne).

[5] Li Z., Li X., Nai S., Geng Q., Liao J., Xu X. (2017). Checkpoint kinase 1-induced phosphorylation of O-linked beta-N-acetylglucosamine transferase regulates the intermediate filament network during cytokinesis. J. Biol. Chem..

[6] Na H.J., Akan I., Abramowitz L.K., Hanover J.A. (2020). Nutrient-driven O-GlcNAcylation controls DNA damage repair signaling and stem/progenitor cell homeostasis. Cell Rep..

[7] Stephen H.M., Adams T.M., Wells L. (2021). Regulating the regulators: mechanisms of substrate selection of the O-GlcNAc cycling enzymes OGT and OGA. Glycobiology.

[8] Ruan H.B., Ma Y., Torres S., Zhang B., Feriod C., Heck R.M. (2017). Calcium-dependent O-GlcNAc signaling drives liver autophagy in adaptation to starvation. Genes Dev..

[9] Kaasik K., Kivimae S., Allen J.J., Chalkley R.J., Huang Y., Baer K. (2013). Glucose sensor O-GlcNAcylation coordinates with phosphorylation to regulate circadian clock. Cell Metab..

[10] Seo H.G., Kim H.B., Kang M.J., Ryum J.H., Yi E.C., Cho J.W. (2016). Identification of the nuclear localisation signal of O-GlcNAc transferase and its nuclear import regulation. Sci. Rep..

[11] Xu Q., Yang C., Du Y., Chen Y., Liu H., Deng M. (2014). AMPK regulates histone H2B O-GlcNAcylation. Nucleic Acids Res..

[12] Bullen J.W., Balsbaugh J.L., Chanda D., Shabanowitz J., Hunt D.F., Neumann D. (2014). Cross-talk between two essential nutrient-sensitive enzymes: O-GlcNAc transferase (OGT) and AMP-activated protein kinase (AMPK). J. Biol. Chem..

[13] Wang Y., Shu H., Liu J., Jin X., Wang L., Qu Y. (2022). EGF promotes PKM2 O-GlcNAcylation by stimulating O-GlcNAc transferase phosphorylation at Y976 and their subsequent association. J. Biol. Chem..

[14] Maze I., Noh K.M., Soshnev A.A., Allis C.D. (2014). Every amino acid matters: essential contributions of histone variants to mammalian development and disease. Nat. Rev. Genet..

[15] Pitasse-Santos P., Hewitt-Richards I., Abeywickrama Wijewardana Sooriyaarachchi M.D., Doveston R.G. (2024). Harnessing the 14-3-3 protein-protein interaction network. Curr. Opin. Struct. Biol..

[16] Zhou T., Aumais J.P., Liu X., Yu-Lee L.Y., Erikson R.L. (2003). A role for Plk1 phosphorylation of NudC in cytokinesis. Dev. Cell.

[17] Madeira F., Tinti M., Murugesan G., Berrett E., Stafford M., Toth R. (2015). 14-3-3-Pred: improved methods to predict 14-3-3-binding phosphopeptides. Bioinformatics.

[18] Soini L., Leysen S., Davis J., Ottmann C. (2022). Molecular glues to stabilise protein-protein interactions. Curr. Opin. Chem. Biol..

[19] Somsen B., Cossar P., Arkin M., Brunsveld L., Ottmann C. (2024). 14-3-3 protein-protein interactions: from mechanistic understanding to their small-molecule stabilization. Chembiochem.

[20] Toleman C.A., Schumacher M.A., Yu S.H., Zeng W., Cox N.J., Smith T.J. (2018). Structural basis of O-GlcNAc recognition by mammalian 14-3-3 proteins. Proc. Natl. Acad. Sci. U. S. A..

[21] Short J.D., Dere R., Houston K.D., Cai S.L., Kim J., Bergeron J.M. (2010). AMPK-mediated phosphorylation of murine p27 at T197 promotes binding of 14-3-3 proteins and increases p27 stability. Mol. Carcinog.

[22] Madigan J.P., Hou F., Ye L., Hu J., Dong A., Tempel W. (2018). The tuberous sclerosis complex subunit TBC1D7 is stabilized by Akt phosphorylation-mediated 14-3-3 binding. J. Biol. Chem..

[23] Seo H.G., Kim H.B., Yoon J.Y., Kweon T.H., Park Y.S., Kang J. (2020). Mutual regulation between OGT and XIAP to control colon cancer cell growth and invasion. Cell Death Dis..

[24] Yang Y., Yin X., Yang H., Xu Y. (2015). Histone demethylase LSD2 acts as an E3 ubiquitin ligase and inhibits cancer cell growth through promoting proteasomal degradation of OGT. Mol. Cell.

[25] Zhang H., Xue K., Li W., Yang X., Gou Y., Su X. (2024). Cullin5 drives experimental asthma exacerbations by modulating alveolar macrophage antiviral immunity. Nat. Commun..

[26] Peng K., Liu R., Jia C., Wang Y., Jeong G.H., Zhou L. (2021). Regulation of O-linked N-Acetyl glucosamine transferase (OGT) through E6 stimulation of the ubiquitin ligase activity of E6AP. Int. J. Mol. Sci..

[27] Meng L., Dong R., Mi W., Qin K., Ouyang K., Sun J. (2024). The ubiquitin E3 ligase APC/C(Cdc20) mediates mitotic degradation of OGT. J. Biol. Chem..

[28] Tang J., Long G., Hu K., Xiao D., Liu S., Xiao L. (2023). Targeting USP8 inhibits O-GlcNAcylation of SLC7A11 to promote ferroptosis of hepatocellular carcinoma via stabilization of OGT. Adv. Sci. (Weinh).

[29] Dey A., Seshasayee D., Noubade R., French D.M., Liu J., Chaurushiya M.S. (2012). Loss of the tumor suppressor BAP1 causes myeloid transformation. Science.

[30] Tang J., Long G., Li X., Zhou L., Zhou Y., Wu Z. (2023). The deubiquitinase EIF3H promotes hepatocellular carcinoma progression by stabilizing OGT and inhibiting ferroptosis. Cell Commun. Signal.

[31] Du Y., Yang Z., Shi H., Chen Z., Chen R., Zhou F. (2024). E3 ubiquitin ligase UBR5 promotes gemcitabine resistance in pancreatic cancer by inducing O-GlcNAcylation-mediated EMT via destabilization of OGA. Cell Death Dis..

[32] Kweon T.H., Jung H., Ko J.Y., Kang J., Kim W., Kim Y. (2024). O-GlcNAcylation of RBM14 contributes to elevated cellular O-GlcNAc through regulation of OGA protein stability. Cell Rep..

